# Supplementary material for: Correlating Basal Gene Expression across Chemical Sensitivity Data to Screen for Novel Synergistic Interactors of HDAC Inhibitors in Pancreatic Carcinoma
Source: Pharmaceuticals (Basel). 2023 Feb 14;16(2):294. doi: 10.3390/ph16020294 (PMC9964546; doi:10.3390/ph16020294)
Supplement: Supplementary file 1 [file pharmaceuticals-16-00294-s001.zip › Supplementary Note S1 Detailed results of experimental evaluation of synergisms with concen-tration annotations.pdf]

SUPPORTING INFORMATION

Correlating basal gene expression across chemical sensitivity data to screen for novel synergistic interactors of HDAC inhibitors in pancreatic carcinoma

Nemanja Djokovic [1, *], Ana Djuric [2], Dusan Ruzic [1], Tatjana Srdic-Rajic [2] and Katarina Nikolic [1,*]

[1] Department of Pharmaceutical Chemistry, Faculty of Pharmacy, University of Belgrade, Vojvode Stepe 450, 11221 Belgrade, Serbia

[2] Department of Experimental Oncology, Institute for Oncology and Radiology of Serbia, Pasterova 14, 11000 Belgrade, Serbia

* Correspondence: nemanja.djokovic@pharmacy.bg.ac.rs (N.D.); katarina.nikolic@pharmacy.bg.ac.rs (K.N.)

Supplementary Note S1. Detailed results of experimental evaluation of synergisms with concentration annotations.

Legend:

**Symbol**	**Description**
+ + + + +	Very strong synergism
+ + +	Synergism
+ +	Moderate synergism
+	Slight synergism
±	Nearly additive
–	Slight antagonism
– –	Moderate antagonism
– – –	Antagonism
– – – –	Strong antagonism
– – – – –	Very strong antagonism

MIA PaCa-2 cell line

	Drug concentration			
Drug	I	II	III	IV
HDACi (**6b**)	48.60±7.45	13.58±2.33	4.80±1.01	2.91±0.87
ROCKi (RKI-1447)	64.18±8.12	40.49±5.55	37.49±3.02	30.84±7.33
**6b** + RKI-1447	83.28±10.34	55.48±7.51	37.69±5.38	38.31±4.56
Combination Index (CI)	0.445	0.981	1.253	0.605
Interaction **6b** + RKI-1447	+ + +	+	– –	+ +

Compound 6 (μM): I=50; II=25; III=12.5; IV=6.25
RKI (μM): I=30; II=15; III=7.5; IV=3.75

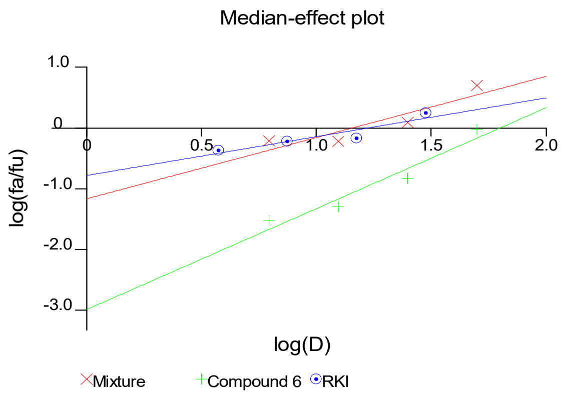

Figure S1. The median-effect plot for tested synergism between 6b and RKI-1447 on MIA PaCa-2 cell line. In this plot, *fa* is the fraction of cells affected, while the *fu* is the fraction of cells unaffected, D is the dose of a drug.

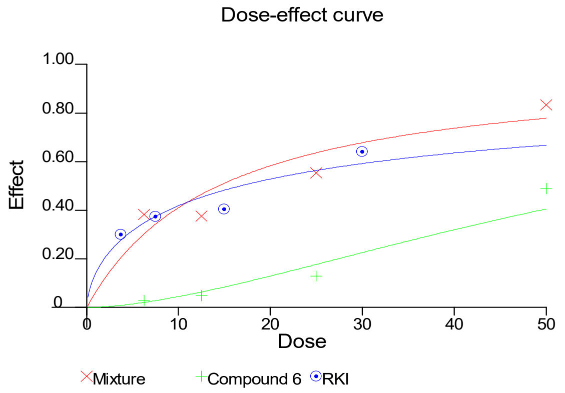

Figure S2. Dose-effect curve for tested synergism between 6b and RKI-1447 on MIA PaCa-2 cell line.

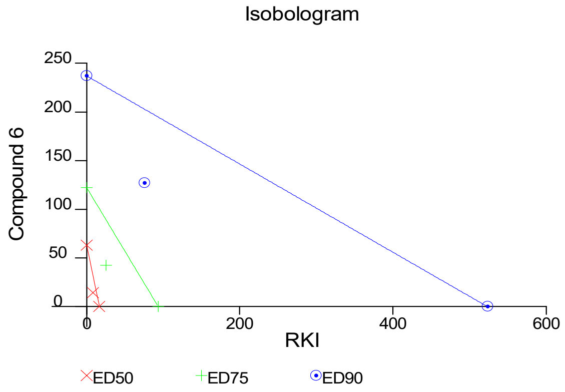

Figure S3. Isobologram for synergism between 6b and RKI-1447 on MIA PaCa-2 cell line.

MIA PaCa-2 cell line

Drug	Drug concentration			
	I	II	III	IV
HDACi (**8b**)	27.30±4.44	1.83±0.70	0.94±0.45	0.57±0.19
ROCKi (RKI-1447)	62.94±10.11	42.93±6.99	36.07±7.51	35.46±4.09
**8b** + RKI-1447	73.02±7.44	45.32±5.14	31.14±5.21	28.91±2.34
Combination Index (CI)	0.333	1.564	2.469	1.505
Interaction **8b** + RKI-1447	+ + +	− − −	− − −	− − −

Compound 8 (μM): I=6; II=3; III=1.5; IV=0.75
RKI (μM): I=30; II=15; III=7.5; IV=3.75

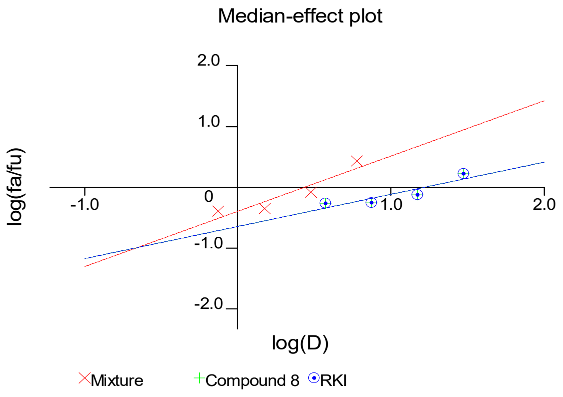

Figure S4. The median-effect plot for tested synergism between 8b and RKI-1447 on MIA PaCa-2 cell line. In this plot, *fa* is the fraction of cells affected, while the *fu* is the fraction of cells unaffected, D is the dose of a drug.

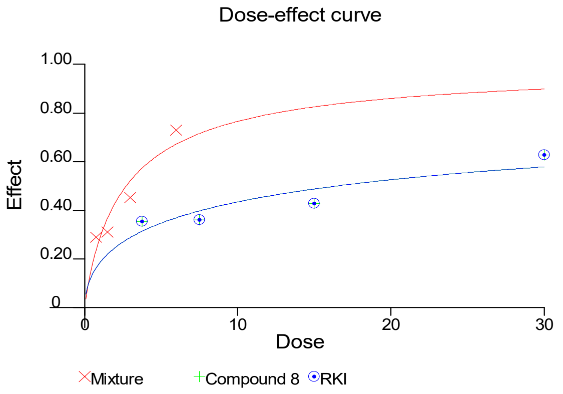

Figure S5. Dose-effect curve for tested synergism between 8b and RKI-1447 on MIA PaCa-2 cell line.

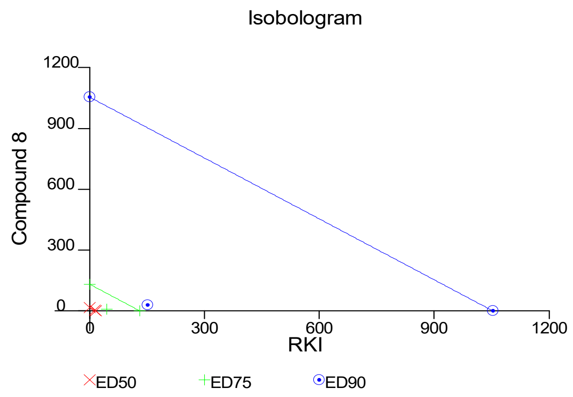

Figure S6. Isobologram for synergism between 8b and RKI-1447 on MIA PaCa-2 cell line.

MIA PaCa-2 cell line

Drug	Drug concentration			
	I	II	III	IV
HDACi (**9b**)	44.65±3.39	18.25±2.22	1.71±0.67	0.46±0.20
ROCKi (RKI-1447)	61.64±7.78	43.28±5.38	35.02±3.25	29.16±2.16
**9b** + RKI-1447	78.03±7.18	64.37±6.78	41.91±5.98	33.32±4.21
Combination Index (CI)	0.513	0.577	0.964	0.803
Interaction **9b** + RKI-1447	+ + +	+ + +	±	+ +

Compound 9 (μM): I=50; II=25; III=12.5; IV=6.25
RKI (μM): I=30; II=15; III=7.5; IV=3.75

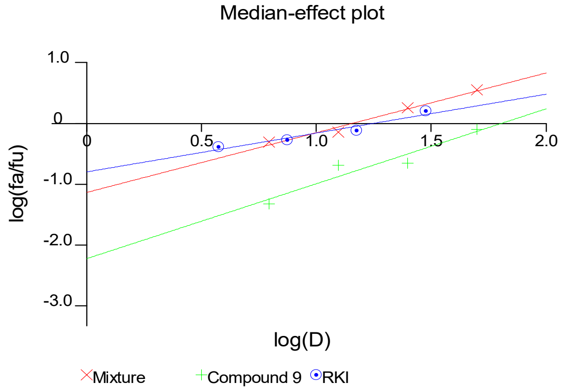

Figure S7. The median-effect plot for tested synergism between 9b and RKI-1447 on MIA PaCa-2 cell line. In this plot, *fa* is the fraction of cells affected, while the *fu* is the fraction of cells unaffected, D is the dose of a drug.

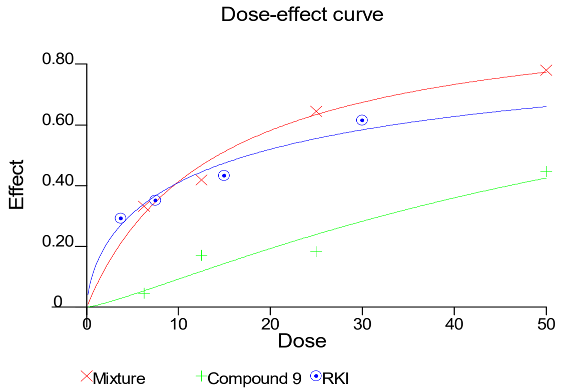

Figure S8. Dose-effect curve for tested synergism between 9b and RKI-1447 on MIA PaCa-2 cell line.

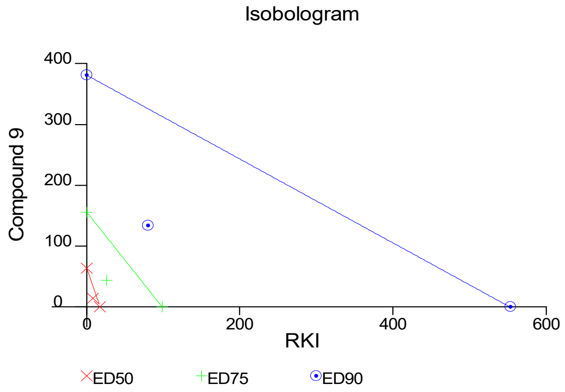

Figure S9. Isobologram for synergism between 9b and RKI-1447 on MIA PaCa-2 cell line.

Panc-1 cell line

Drug	Drug concentration I	II	III	IV
HDACi (**6b**)	23.61±3.26	1.39±0.55	0.47±0.21	0.5±0.2
ROCKi (RKI-1447)	62.76±11.23	33.89±8.92	22.02±7.22	18.11±5.90
**6b** + RKI-1447	69.79±10.45	48.51±6.34	24.98±3.13	8.92±4.21
Combination Index (CI)	0.992	1.050	1.334	2.117
Interaction **6b** + RKI-1447	±	±	− −	− − −

Compound 6 (μM): I=50; II=25; III=12.5; IV=6.25
RKI (μM): I=50; II=25; III=12.5; IV=6.25

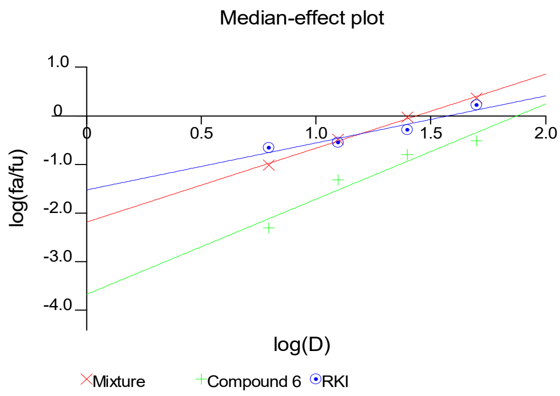

Figure S10. The median-effect plot for tested synergism between 6b and RKI-1447 on Panc-1 cell line. In this plot, *fa* is the fraction of cells affected, while the *fu* is the fraction of cells unaffected, D is the dose of a drug.

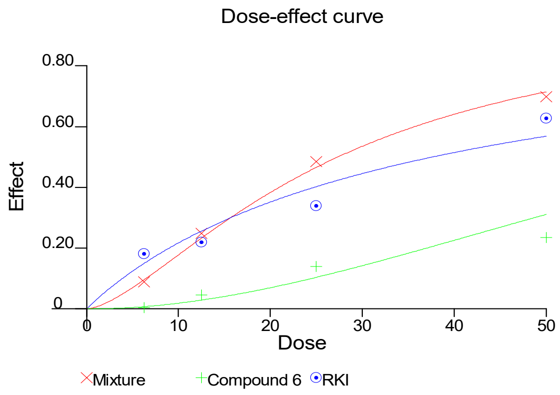

Figure S11. Dose-effect curve for tested synergism between 6b and RKI-1447 on Panc-1 cell line.

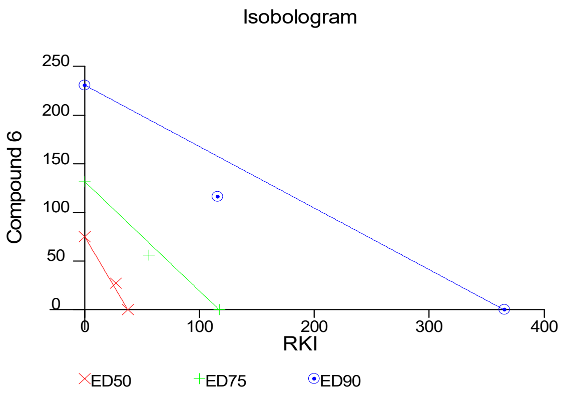

Figure S12. Isobologram for synergism between 6b and RKI-1447 on Panc-1 cell line.

Panc-1 cell line

Drug	Drug concentration			
	I	II	III	IV
HDACi (**8b**)	49.78±6.77	38.21±5.89	17.13±2.11	0.1±0.05
ROCKi (RKI-1447)	58.78±5.55	30.77±3.45	17.27±2.28	7.41±2.56
**8b** + RKI-1447	63.94±6.33	42.96±5.79	21.99±3.45	6.79±3.06
Combination Index (CI)	3.133	1.433	1.266	1.474
Interaction **8b** + RKI-1447	– – –	– –	– –	– – –

Compound 8 (μM): I=20; II=10; III=5.0; IV=2.5
RKI (μM): I=50; II=25; III=12.5; IV=6.25

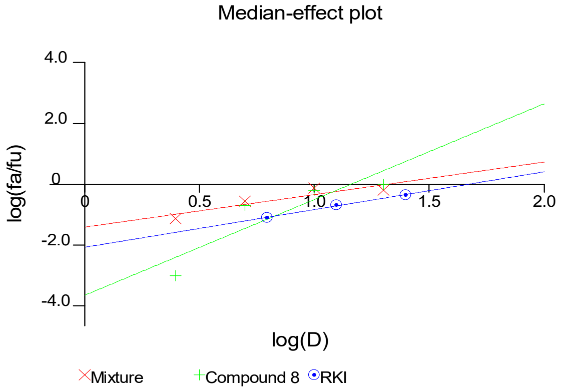

Figure S13. The median-effect plot for tested synergism between 8b and RKI-1447 on Panc-1 cell line. In this plot, *fa* is the fraction of cells affected, while the *fu* is the fraction of cells unaffected, D is the dose of a drug.

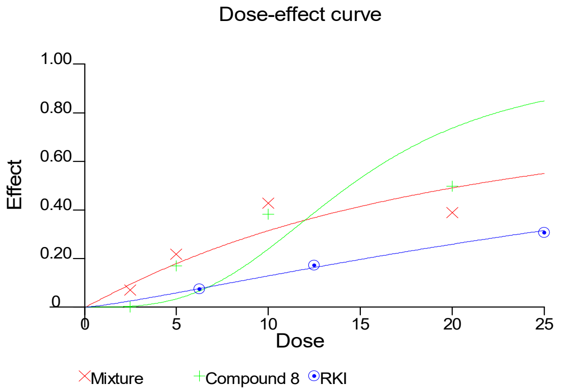

Figure S14. Dose-effect curve for tested synergism between 8b and RKI-1447 on Panc-1 cell line.

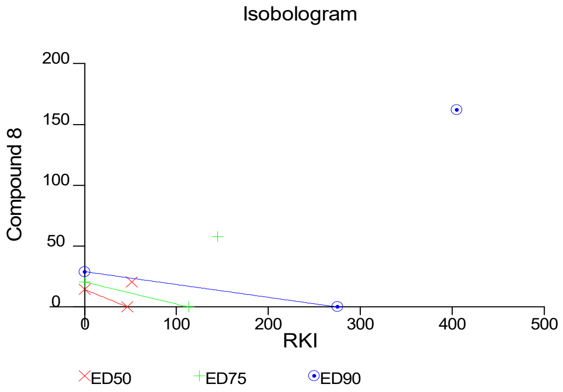

Figure S15. Isobologram for synergism between 8b and RKI-1447 on Panc-1 cell line.

Panc-1 cell line

	Drug concentration			
Drug	I	II	III	IV
HDACi (**9b**)	45.18±4.55	23.14±7.42	7.56±3.71	1.12±0.89
ROCKi (RKI-1447)	64.82±6.41	37.92±5.34	19.76±2.30	9.41±1.06
**9b** + RKI-1447	64.68±5.01	51.26±7.20	33.14±6.10	18.01±4.02
Combination Index (CI)	1.705	1.207	0.975	0.823
Interaction **9b** + RKI-1447	− − −	− −	±	+

Compound 9 (μM): I=50; II=25; III=12.5; IV=6.25
RKI (μM): I=50; II=25; III=12.5; IV=6.25

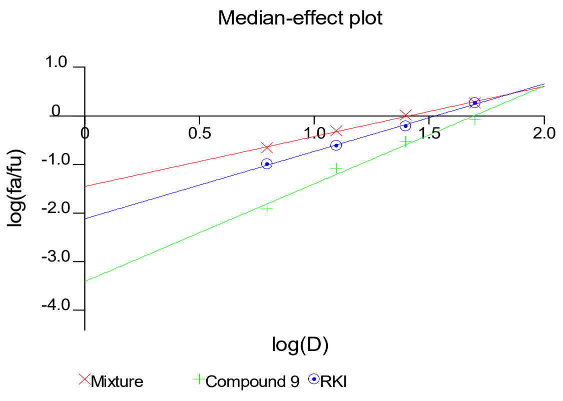

Figure S16. The median-effect plot for tested synergism between 9b and RKI-1447 on Panc-1 cell line. In this plot, *fa* is the fraction of cells affected, while the *fu* is the fraction of cells unaffected, D is the dose of a drug.

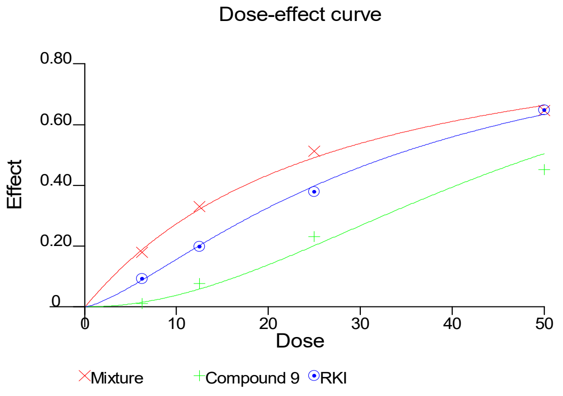

Figure S17. Dose-effect curve for tested synergism between 9b and RKI-1447 on Panc-1 cell line.

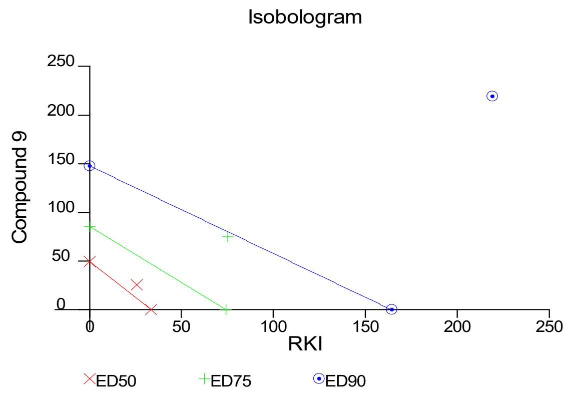

Figure S18. Isobologram for synergism between 9b and RKI-1447 on Panc-1 cell line.

MIA PaCa-2 cell line

Drug	Drug concentration			
	I	II	III	IV
HDACi (**6b**)	71.49±11.91	41.62±11.91	23.06±9.75	16.27±5.34
Fingolimod	79.98±10.36	48.31±12.04	20.57±4.85	20.33±5.37
**6b** + Fingolimod	89.28±4.05	60.81±20.19	34.11±14.58	26.96±7.59
Combination Index (CI)	0.780	1.422	1.648	1.070
Interaction **6b** + Fingolimod	+ +	– –	– – –	±

Compound 6 (μM): I=50; II=25; III=12.5; IV=6.25
Fingolimod (μM): I=8; II=4; III=2; IV=1

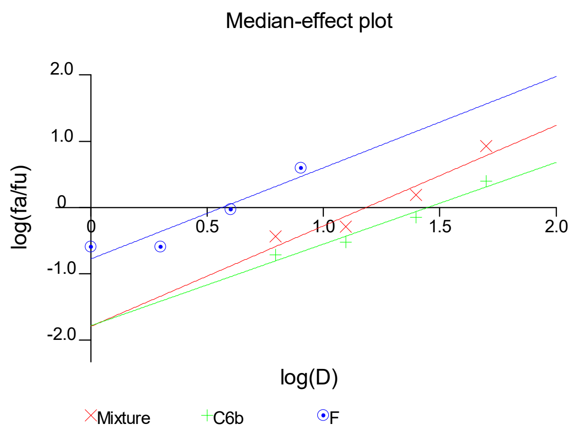

Figure S19. The median-effect plot for tested synergism between 6b and fingolimod on MIA PaCa-2 cell line. In this plot, *fa* is the fraction of cells affected, while the *fu* is the fraction of cells unaffected, D is the dose of a drug.

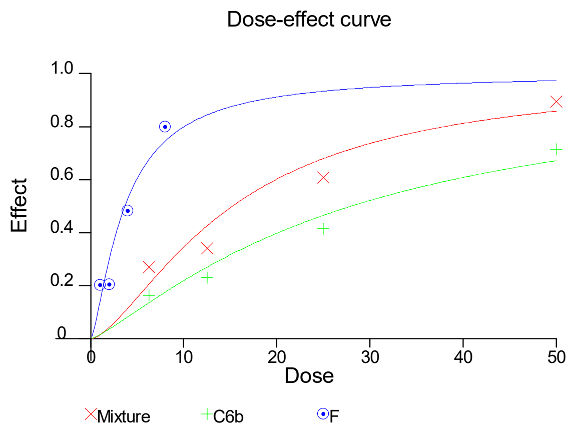

Figure S20. Dose-effect curve for tested synergism between 6b and fingolimod on MIA PaCa-2 cell line.

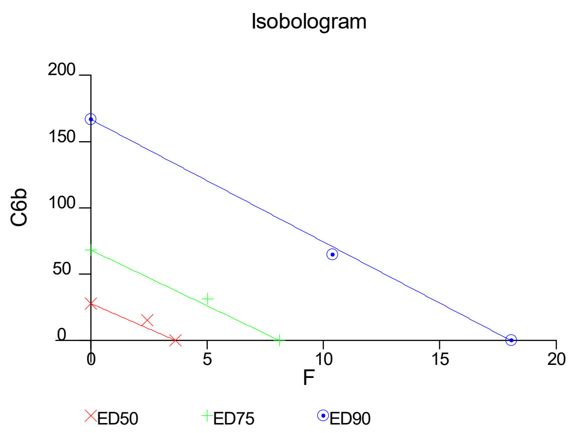

Figure S21. Isobologram for synergism between 6b and fingolimod on MIA PaCa-2 cell line.

MIA PaCa-2 cell line

Drug	Drug concentration			
	I	II	III	IV
HDACi (**8b**)	81.22±0.96	68.72±2.69	36.08±3.29	16.72±4.28
Fingolimod	87.22±2.01	52.02±2.47	23.73±3.92	22.83±1.89
**8b** + Fingolimod	91.31±10.08	65.06±1.92	38.98±5.26	26.55±2.94
Combination Index (CI)	1.188	1.836	1.852	1.338
Interaction **8b** + Fingolimod	– – –	– – –	– – –	– –

Compound 8 (μM): I=10; II=5; III=2.5; IV=1.25
Fingolimod (μM): I=8; II=4; III=2; IV=1

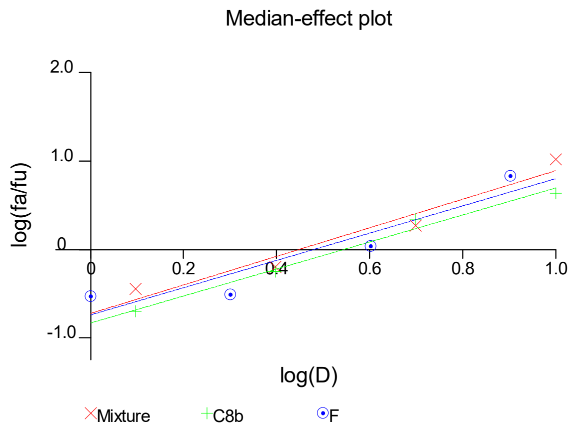

Figure S22. The median-effect plot for tested synergism between 8b and fingolimod on MIA PaCa-2 cell line. In this plot, *fa* is the fraction of cells affected, while the *fu* is the fraction of cells unaffected, D is the dose of a drug.

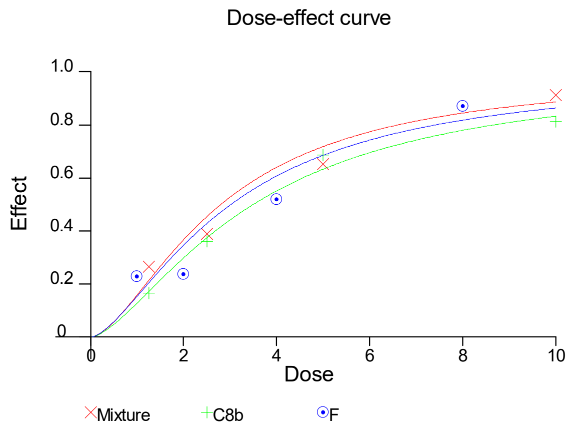

Figure S23. Dose-effect curve for tested synergism between 8b and fingolimod on MIA PaCa-2 cell line.

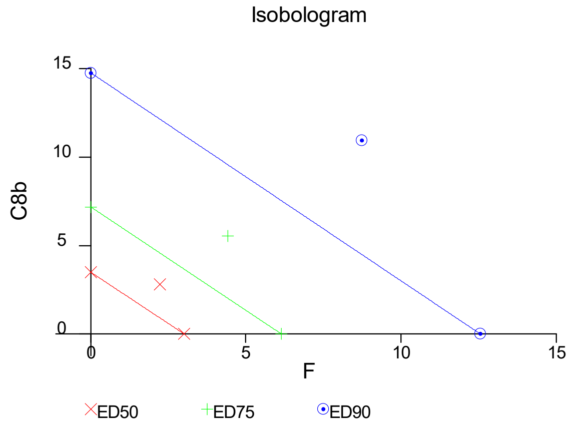

Figure S24. Isobologram for synergism between 8b and fingolimod on MIA PaCa-2 cell line.

MIA PaCa-2 cell line

	Drug concentration			
Drug	I	II	III	IV
HDACi (**9b**)	83.61±0.27	69.42±11.34	53.31±17.80	44.84±11.21
Fingolimod	76.58±4.48	63.82±6.81	43.78±12.65	35.34±6.99
**9b** + Fingolimod	89.11±1.57	73.59±12.40	55.57±18.16	47.22±9.51
Combination Index (CI)	0.870	1.449	1.773	1.293
Interaction **9b** + Fingolimod	+	– –	– – –	– –

Compound 9 (μM): I=50; II=25; III=12.5; IV=6.25
Fingolimod (μM): I=8; II=4; III=2; IV=1

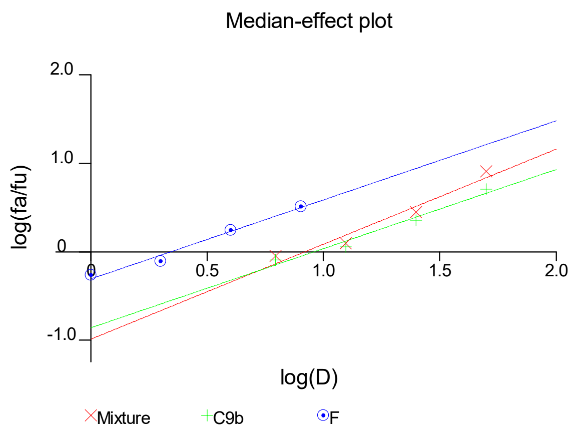

Figure S25. The median-effect plot for tested synergism between 9b and fingolimod on MIA PaCa-2 cell line. In this plot, *fa* is the fraction of cells affected, while the *fu* is the fraction of cells unaffected, D is the dose of a drug.

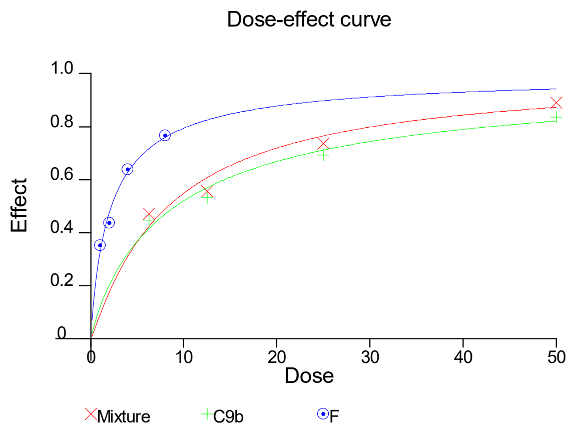

Figure S26. Dose-effect curve for tested synergism between 9b and fingolimod on MIA PaCa-2 cell line.

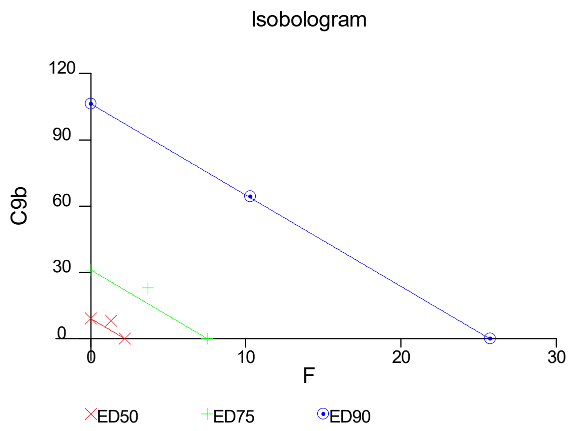

Figure S27. Isobologram for synergism between 9b and fingolimod on MIA PaCa-2 cell line.

Panc-1 cell line

Drug	Drug concentration			
	I	II	III	IV
HDACi (**6b**)	63.68±4.70	25.29±6.71	23.93±6.23	10.30±1.96
Fingolimod	79.18±1.45	40.09±4.97	24.56±1.24	18.99±0.95
**6b** + Fingolimod	84.96±2.05	67.22±5.98	37.87±5.71	21.98±0.78
Combination Index (CI)	0.847	0.945	1.232	1.141
Interaction **6b** + Fingolimod	+ +	±	− −	−

Compound 6 (μM): I=100; II=50; III=25; IV=12.5
Fingolimod (μM): I=12; II=6; III=3; IV=1.5

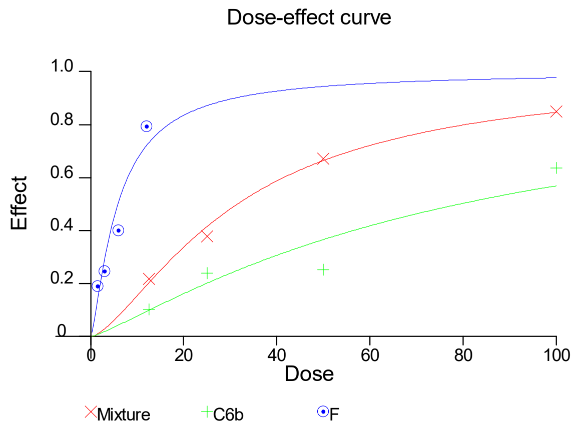

Figure S28. The median-effect plot for tested synergism between 6b and fingolimod on Panc-1 cell line. In this plot, *fa* is the fraction of cells affected, while the *fu* is the fraction of cells unaffected, D is the dose of a drug.

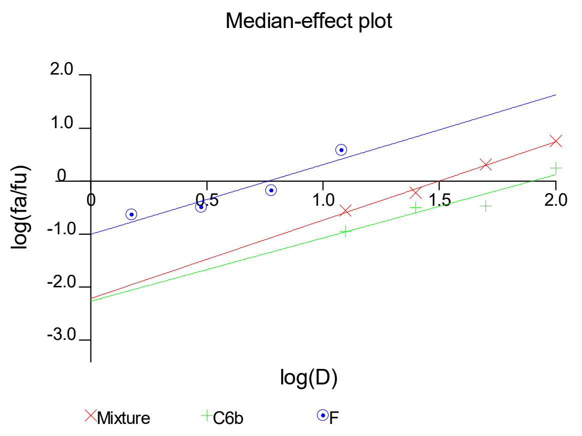

Figure S29. Dose-effect curve for tested synergism between 6b and fingolimod on Panc-1 cell line.

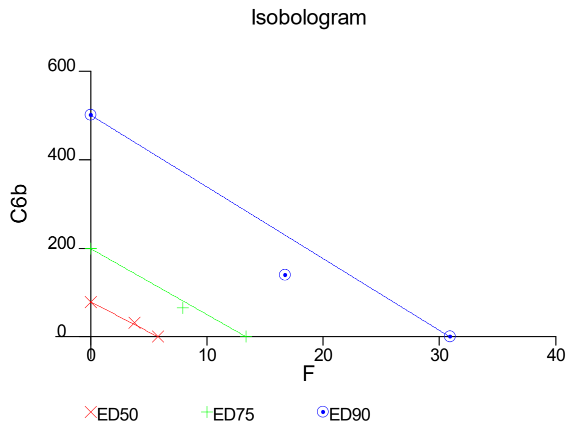

Figure S30. Isobologram for synergism between 6b and fingolimod on Panc-1 cell line.

Panc-1 cell line

	Drug concentration			
Drug	I	II	III	IV
HDACi (**8b**)	58.10±0.24	23.86±9.24	4.77±2.69	4.68±2.33
Fingolimod	68.24±4.62	41.81±5.37	19.44±10.97	12.27±8.75
**8b** + Fingolimod	74.74±5.61	30.91±6.84	16.60±7.22	9.42±5.57
Combination Index (CI)	1.267	1.806	2.027	1.592
Interaction **8b** + Fingolimod	– –	– – –	– – – –	– – –

Compound 8 (μM): I=20; II=10; III=5.0; IV=2.5
Fingolimod (μM): I=12; II=6; III=3; IV=1.5

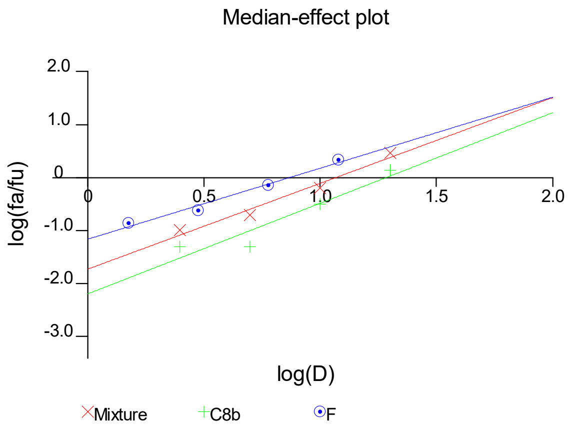

Figure S31. The median-effect plot for tested synergism between 8b and fingolimod on Panc-1 cell line. In this plot, *fa* is the fraction of cells affected, while the *fu* is the fraction of cells unaffected, D is the dose of a drug.

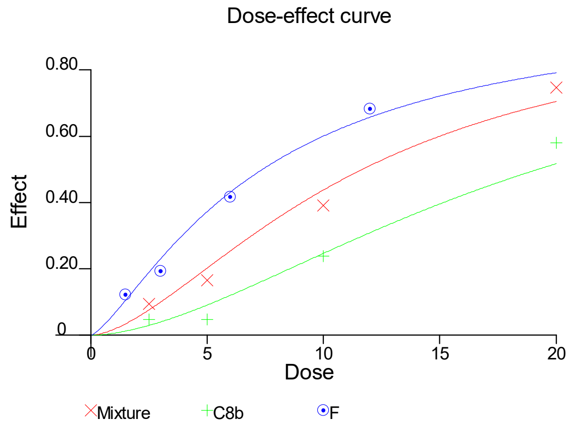

Figure S32. Dose-effect curve for tested synergism between 8b and fingolimod on Panc-1 cell line.

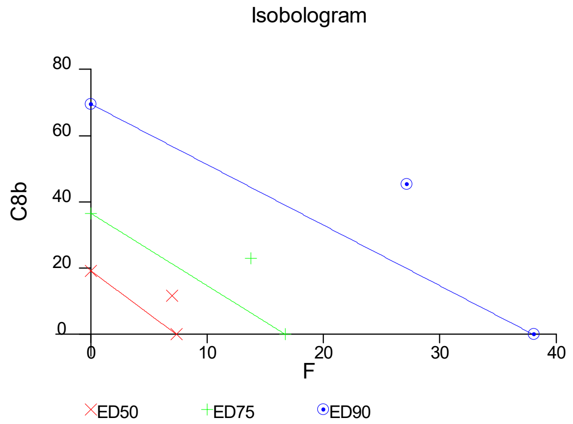

Figure S33. Isobologram for synergism between 8b and fingolimod on Panc-1 cell line.

Panc-1 cell line

Drug	Drug concentration			
	I	II	III	IV
HDACi (**9b**)	61.28±6.91	50.93±3.83	40.71±3.74	24.03±5.73
Fingolimod	62.10±4.94	50.04±3.20	31.21±11.85	21.17±3.33
**9b** + Fingolimod	76.50±5.14	66.12±10.76	53.41±18.12	33.34±10.07
Combination Index (CI)	0.911	0.848	0.810	1.120
Interaction **9b** + Fingolimod [b]	±	+ +	+ +	–

Compound 9 (μM): I=50; II=25; III=12.5; IV=6.25
Fingolimod (μM): I=12; II=6; III=3; IV=1.5

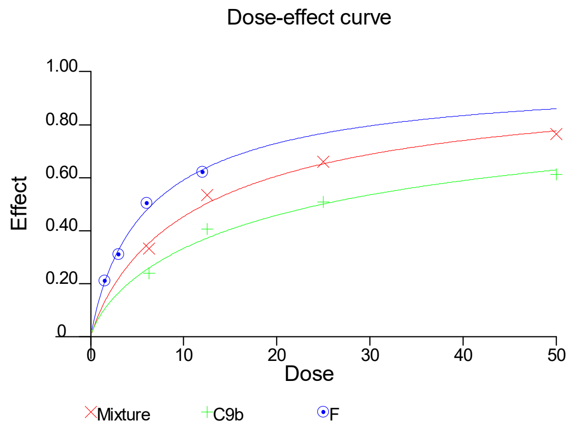

Figure S34. The median-effect plot for tested synergism between 9b and fingolimod on Panc-1 cell line. In this plot, *fa* is the fraction of cells affected, while the *fu* is the fraction of cells unaffected, D is the dose of a drug.

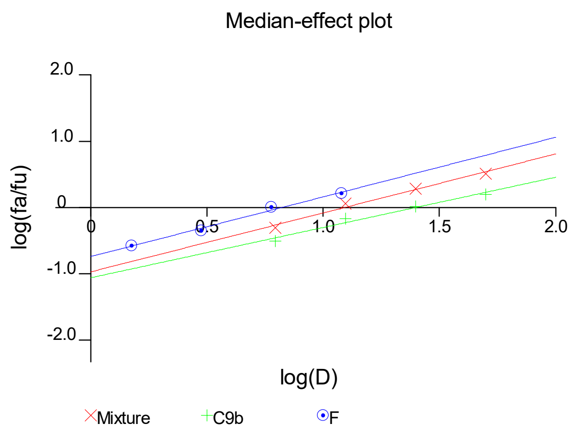

Figure S35. Dose-effect curve for tested synergism between 9b and fingolimod on Panc-1 cell line.

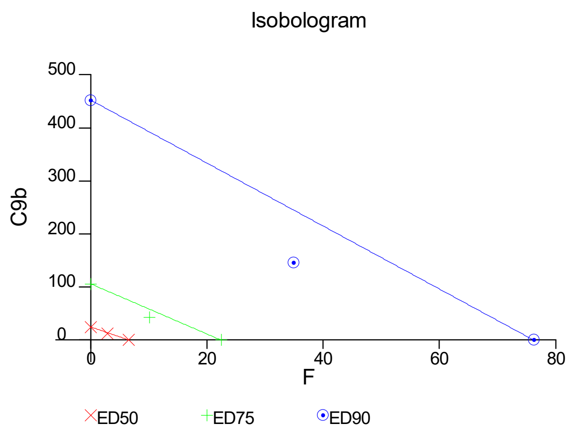

Figure S36. Isobologram for synergism between 9b and fingolimod on Panc-1 cell line.
